# *In vivo* evaluation of a chitosan/PLGA/chitosan composite versus monolayer PLGA electrospun scaffold for dorsal onlay urethroplasty: a controlled study in rabbits

**DOI:** 10.3389/fbioe.2026.1891449

**Published:** 2026-10-19

**Authors:** Marco Bassam Mahdi, Fatemeh Mohtaram, Rikke Nørresø Haase, Grazvydas Tuckus, Peter Fojan, Joar Bergman, Christian Thomsen, Benedict Kjærgaard, Sanne Ross Sørensen, Hiva Alipour, Tariq O. Abbas, Cristian Pablo Pennisi, Tommy Kjærgaard Nielsen

**Affiliations:** 1 Department of Renal Surgery and Urology, Aalborg University Hospital, Aalborg, Denmark; 2 Department of Clinical Medicine, Aalborg University, Aalborg, Denmark; 3 Materials Science and Engineering Group, Department of Materials and Production, Aalborg University, Aalborg, Denmark; 4 Department of Pathology, Aalborg University Hospital, Aalborg, Denmark; 5 Department of Cardiothoracic Surgery, Aalborg University Hospital, Aalborg, Denmark; 6 Biomedical Research Laboratory, Aalborg University Hospital, Aalborg, Denmark; 7 Global Andrology Forum, Global Andrology Foundation, Moreland Hills, OH, United States; 8 Department of Health Science and Technology, Aalborg University, Aalborg, Denmark; 9 Department of Pediatric Urology, Sidra Medicine and Weill Cornell Medical College, Doha, Qatar; 10 Regenerative Medicine, Department of Health Science and Technology, Faculty of Medicine, Aalborg University, Aalborg, Denmark

**Keywords:** angiogenesis, chitosan, electrospun scaffold, foreign-body response, PLGA, rabbit model, tissue engineering, urethroplasty

## Abstract

**Background:**

Autologous buccal mucosa grafts remain the surgical standard for complex urethral stricture repair but are limited by donor-site morbidity, restricted tissue availability, and inadequate graft vascularization. Acellular scaffolds, whether synthetic or natural, can offer either mechanical or biological signals but not both. Hybrid composite scaffolds combining synthetic structural support with natural bioactive polymers have been proposed to address this limitation. In this work, a trilayer scaffold consisting of a poly(lactide-co-glycolide) (PLGA) core with chitosan on either side was assessed *in vivo* in a controlled rabbit dorsal onlay urethroplasty model.

**Methods:**

Twenty-four male New Zealand White rabbits were allocated 1:1:1 to dorsal onlay urethroplasty with the trilayer scaffold, a monolayer PLGA scaffold, or sham control, using weight-stratified allocation. The primary endpoint was vessel density on CD31-stained sections at 12 weeks; secondary endpoints included tissue thickness, fibrosis, smooth muscle content, re-epithelialization, inflammatory cell scores, and urethrography, assessed by blinded observers.

**Results:**

Vessel density in the trilayer was intermediate between sham (highest) and the monolayer (lowest), with significant overall between-group variation (*p* = 0.008); the trilayer did not significantly differ from the monolayer (*p* = 0.248). Macrophage scores showed a non-significant trend toward lower values in the trilayer than in the monolayer (*p* = 0.062), and multinucleated giant-cell scores were significantly higher in both scaffold groups than in sham (*p* = 0.026), consistent with a foreign-body response. Vessel density was inversely correlated with tissue thickness and fibrosis, defining a coherent host-response signature. Re-epithelialization was complete across groups without ulceration features on histological review. Retrograde urethrography demonstrated preserved patency in all 23 animals completing follow-up, with no ≥50% stricture observed.

**Conclusion:**

The trilayer scaffold was feasible to implant and well tolerated in a rabbit dorsal onlay urethroplasty model, supporting tissue regeneration with preserved urethral patency. The trilayer did not significantly increase vessel density over the monolayer at 12 weeks, although a non-significant trend toward lower macrophage scores was observed in the trilayer group. These findings refine rather than confirm the development hypothesis and motivate future studies with earlier endpoints, mechanical-modulus tuning, and alternative polymer ratios.

## 1 Introduction

Despite three decades of surgical refinement, urethral stricture disease remains a clinical challenge. Buccal mucosa graft urethroplasty, the current definitive treatment, is followed by re-stricture in approximately one in five patients within 4 years (Spilotros et al., 2017), with inadequate graft vascularization a recognized contributor to failure (Hampson et al., 2014). It is further constrained by donor-site morbidity and limited tissue availability (Barbagli et al., 2010; Desai et al., 2025). These limitations motivate the development of pre-fabricated urethral substitutes capable of supporting host vascularization, with parallel application in pediatric hypospadias surgery where proximal or reoperative repairs require urethral plate substitution (Tsachouridis et al., 2023).

Synthetic electrospun scaffolds represent one such strategy. They can be fabricated with a fibrous architecture resembling native extracellular matrix and tunable mechanical properties suited to surgical handling. However, purely synthetic polymer scaffolds such as poly(L-lactide) (PLA) typically show limited biocompatibility and a reduced capacity to promote the early vascularization required for graft integration (Abbas et al., 2024). Bulk hydrolytic degradation also produces acidic by-products that can amplify the foreign-body response *in vivo* (Ji et al., 2012).

Natural polymer scaffolds occupy the complementary end of the trade-off. They include decellularized extracellular matrices such as small intestinal submucosa (SIS) and reconstituted polymers such as collagen and chitosan, which retain bioactive cues that promote cellular attachment and proliferation but are typically constrained by inferior mechanical properties, batch-to-batch variability, and inconsistent host integration; the performance of these scaffolds in urethral tissue engineering has been reviewed in detail elsewhere (Žiaran et al., 2017; Abbas et al., 2019).

This complementarity motivates composite scaffolds that combine synthetic structural support with natural bioactive polymers. Chitosan, a naturally derived polysaccharide, confers hydrophilicity, antimicrobial activity, and pro-angiogenic properties that complement the structural advantages of synthetic polyester cores such as PLA (Dash et al., 2011). Chitosan oligosaccharides drive macrophage polarization toward the M2 phenotype and promote secretion of pro-angiogenic factors including vascular endothelial growth factor (VEGF), supporting early tissue vascularization (Huang et al., 2020). A composite design in which chitosan layers surround a synthetic polyester core may therefore attenuate the foreign-body response and support vascularization while preserving mechanical integrity for surgical use.

The trilayer chitosan/PLGA/chitosan (CPC) composite was designed as a biomimetic construct intended to recapitulate the native layered architecture of the urethral wall (De Graaf et al., 2018). The luminal chitosan layer is positioned to mimic the urethral mucosa, providing a hydrophilic, bioactive surface that supports epithelial cell attachment and migration and contributes antimicrobial properties characteristic of chitosan-based biomaterials (Dash et al., 2011; Abbas et al., 2024). The central electrospun PLGA layer serves as the mechanical backbone, analogous to the submucosal subepithelial tissue, providing tunable tensile strength and gradual hydrolytic degradation over the healing timeframe (Makadia and Siegel, 2011). The outer chitosan layer interfaces with the surrounding corpus spongiosum, promoting cellular infiltration and angiogenesis (Abbas et al., 2024) alongside chitosan-driven macrophage polarization toward an anti-inflammatory M2 phenotype (Huang et al., 2020).

Contemporary work has similarly explored multilayered architectures for urethral repair; Yang et al. (2025) recently reported a trilayer hydrogel construct with distinct anti-fouling, regenerative, and structural layers designed for scarless urethral healing, illustrating a growing recognition that layered scaffold designs may better recapitulate urethral wall complexity than monolithic constructs.

Building on this rationale, Abbas et al. recently characterized a trilayer chitosan/poly(L-lactide)/chitosan composite scaffold produced by sequential electrospinning. *In vitro*, the construct demonstrated superior mechanical strength, suture retention, smooth muscle cell growth, and angiogenic potential compared with monolayer PLA scaffolds (Abbas et al., 2024), motivating *in vivo* evaluation. We adapted this trilayer concept, substituting poly(L-lactide-co-glycolide) (PLGA) for PLA in the core layer. PLGA’s degradation rate, tunable through the lactide:glycolide ratio, offers more flexibility than the slower-degrading PLA, and its clinical precedent in absorbable medical devices supports future translational pathways. The resulting chitosan/PLGA/chitosan composite scaffold is hereafter referred to as CPC.

*In vivo* evaluation of any construct depends not only on the construct itself but on the rigour of the study design. Preclinical urethral tissue engineering has produced a growing number of scaffold candidates, and several *in vivo* rabbit urethroplasty studies have demonstrated the feasibility of scaffold-based reconstruction (Wan et al., 2020; Amesty et al., 2021). However, methodological rigour across this literature has been inconsistent. A 2021 systematic review of 28 rabbit studies published between 2014 and 2020 reported a mean adherence of 53% to the Animal Research: Reporting of *In Vivo* Experiments (ARRIVE) guidelines, with no included study reporting sample-size estimation methodology and the majority lacking blinded outcome assessment (Abbas et al., 2021). This methodological heterogeneity may contribute to the limited translation of promising preclinical findings into clinical use (Chepelova et al., 2024).

We report a controlled *in vivo* evaluation of the CPC scaffold as an acellular construct in the rabbit dorsal onlay urethroplasty model, which mirrors adult substitution urethroplasty technique and is relevant to both stricture and hypospadia preclinical research. The study was designed and reported in accordance with ARRIVE 2.0 guidelines (Percie du Sert et al., 2020), directly addressing each of these documented gaps with an *a priori* sample-size calculation, a prespecified primary endpoint, blinded outcome assessment, and a three-arm comparison against monolayer PLGA and surgical sham. The primary endpoint was vessel density on CD31 immunohistochemistry at 12 weeks; secondary endpoints included tissue thickness, fibrosis, smooth muscle content, re-epithelialization, inflammatory response, and urethral patency. Prior preclinical urethroplasty studies have largely relied on binary or categorical outcomes such as fistula formation, stricture recurrence, and gross patency, which constrain formal power calculation and contribute to the rigor gap identified above. We therefore prespecified a continuous quantitative primary endpoint, vessel density by CD31 immunohistochemistry at 12 weeks, enabling *a priori* sample-size justification, weight-stratified allocation, and blinded outcome assessment in accordance with ARRIVE 2.0.

We hypothesized that the trilayer composite would yield greater vessel density than monolayer PLGA at 12 weeks, consistent with chitosan-driven M2 macrophage polarization and downstream pro-angiogenic signaling reported in prior work (Huang et al., 2020).

## 2 Materials and methods

### 2.1 Ethics statement

All animal experiments were conducted in accordance with Directive 2010/63/EU on the protection of animals used for scientific purposes and approved by the Danish Animal Experiments Inspectorate (Dyreforsøgstilsynet) under license number 2019-15-0201-00339. Experiments were carried out at Aalborg University Hospital and the Biomedical Research Laboratory, Aalborg University Hospital, under supervision of personnel with FELASA Category B or higher certification. All procedures were classified as moderate severity. Humane endpoints were predefined and included anorexia, apathy, weight loss exceeding 20%, signs of pain or distress unresponsive to treatment, fever above 40.5 °C, or reduced consciousness. Animals meeting these criteria were euthanized without delay. At the planned study endpoint (12 weeks), animals were euthanized by intravenous overdose of pentobarbital sodium (140 mg/kg) in accordance with AVMA Guidelines for the Euthanasia of Animals (Leary et al., 2020). The study is reported in accordance with the ARRIVE 2.0 guidelines for the reporting of animal research (Percie du Sert et al., 2020). The study protocol, including hypothesis, design features, sample-size justification, and analysis plan, was prepared in advance of study commencement; however, it was not pre-registered in a public protocol registry.

### 2.2 Scaffold fabrication

Electrospun scaffolds were fabricated as either a trilayer composite scaffold consisting of chitosan/poly(L-lactide-co-glycolide)/chitosan (CPC) or a monolayer scaffold consisting of poly(L-lactide-co-glycolide) (PLGA) alone. The fabrication protocol was adapted from previously described hybrid scaffold strategies for urethral tissue engineering, with modifications including substitution of TFE as solvent for PLGA and ethanol-based post-processing (Abbas et al., 2024).

PLGA (Resomer® LG 857S, Evonik Industries, Essen, Germany; poly(L-lactide-co-glycolide), ester-terminated, lactide:glycolide 85:15, inherent viscosity 5.0–7.0 dL/g in 0.1% chloroform at 25 °C) was dissolved at 10% (w/v) in 2,2,2-trifluoroethanol (TFE; Sigma-Aldrich, ≥99%) to obtain a homogenous spinning solution. Chitosan (medium molecular weight, catalog 448877, Sigma-Aldrich, St. Louis, MO, United States; CAS 9012-76-4) was dissolved at 2% (w/v) in a mixture of acetic acid, dichloromethane, and distilled water (4:5:1), while polyethylene oxide (PEO; 2% w/v) was prepared in acetic acid and distilled water (1:1). The chitosan and PEO solutions were combined at a 60:40 ratio to improve spinnability, and cetyltrimethylammonium bromide (CTAB; 40 mM) was added as a surfactant.

Electrospinning was performed using a Yflow Electrospinner 2.2D-500 system (Yflow Nanotechnology Solutions) onto a grounded flat collector covered with aluminium foil under ambient laboratory conditions (temperature 22 °C–26 °C; relative humidity 60%–75%, not actively controlled).

For PLGA spinning, the solution was delivered at a flow rate of 1.75 mL/h using a heated syringe pump (26 °C), with an applied voltage of +15.96 kV at the injector, 0 kV at the collector, and a needle-to-collector distance of 17 cm. For chitosan/PEO layers, electrospinning was performed at a flow rate of 2.5 mL/h, with an applied voltage of +22.2 kV at the injector and −17.85 kV at the collector, and a needle-to-collector distance of 17 cm.

Trilayer CPC scaffolds were fabricated by first electrospinning the PLGA core layer, followed by deposition of a chitosan/PEO layer on one side. The scaffold was then flipped, and a second layer of chitosan/PEO was spun to the opposite side to obtain a symmetric trilayer structure.

Scaffold thickness was controlled by electrospinning duration. PLGA layers were produced over 60 min; chitosan layers were applied for 15 min per side. Scaffold morphology and thickness were subsequently assessed on representative samples by scanning electron microscopy using Zeiss EVO 60 (Carl Zeiss Microscopy GmbH, Oberkochen, Germany; see Section 3.1). Fiber diameter was measured manually in ImageJ (NIH, Bethesda, MD, United States) on high-magnification SEM images by drawing a line perpendicular to the fiber axis on 50 randomly selected single foreground fibers per scaffold type.

Following fabrication, scaffolds were washed in deionized water to remove residual polyethylene oxide and subsequently immersed in 70% ethanol for sterilization and neutralization (Lemma et al., 2016). This step also acted as a liquid-leaching extraction process to remove trace residues of organic solvents from the fiber matrix (D’Amato et al., 2017). Scaffolds were then air-dried at room temperature for at least 24 h prior to implantation. Scaffolds were cut to a standard size of 10 × 3 mm before implantation.

### 2.3 Study design and experimental groups

This study was designed as a controlled *in vivo* experimental study to evaluate tissue regeneration following dorsal onlay urethroplasty using synthetic scaffolds. A total of 24 male New Zealand White rabbits were included and allocated in a 1:1:1 ratio to one of three groups: (i) trilayer chitosan/PLGA/chitosan composite scaffold (CPC), (ii) monolayer PLGA scaffold, and (iii) sham (surgical control) without scaffold implantation. The sham group represented unsupported defect healing and served as the reference against which scaffold-mediated effects on remodeling and host response were evaluated.

Experiments were conducted in two enrolment periods, with equal representation of each treatment group within each batch. All animals underwent a standardized surgical procedure with creation of a dorsal urethral defect. In scaffold-treated groups, the defect was reconstructed using the assigned scaffold, whereas in the sham group the urethral tissue was re-approximated without graft implantation.

The primary endpoint was vessel density, assessed on CD31-stained histological sections at 12 weeks post-intervention. Secondary endpoints included tissue thickness, fibrosis, smooth muscle content, re-epithelialization, inflammatory response, and urethral patency assessed by post-mortem urethrography.

Allocation was concealed from all personnel except the principal investigator, who maintained the allocation list. Surgeons were blinded to group allocation during urethral exposure and defect creation. Although CPC and PLGA scaffolds differ in nominal thickness, the two materials were visually and tactilely similar at the time of implantation. Both were thin, white, semi-flexible electrospun sheets that were not reliably distinguishable on inspection or handling. Surgeons therefore remained effectively blinded to scaffold type throughout the procedure. Animal caretakers, veterinarians, and histopathologists were blinded to group allocation throughout the study, and slides were anonymized prior to histological evaluation. Retrograde urethrography was performed and assessed by the principal investigator on anonymized images using study identifiers; the allocation list was not consulted during image evaluation, and group allocation was unmasked only after assessment was complete. Where surgeons and histopathologists were informally asked to guess scaffold type post-implantation, retrospective misidentification was common, supporting the integrity of blinding to scaffold type.

Inclusion criteria comprised male New Zealand White rabbits aged 15–17 weeks, weighing 2.5–3.5 kg, in good general health at completion of the acclimatisation period. Exclusion criteria comprised any animal showing clinical signs of disease, abnormal behaviour, or weight loss exceeding 10% during acclimatisation. No animals met exclusion criteria during acclimatization.

### 2.4 Animals, housing and husbandry

Male New Zealand White rabbits (*Oryctolagus cuniculus*; Charles River Laboratories, L’Arbresle, France) were used in this study. At the time of intervention, animals were aged 15–17 weeks and weighed 2.5–3.5 kg. Upon arrival, animals were identified using ear markings and subcutaneous microchips.

All animals underwent an acclimatization period of 10–14 days prior to intervention. Animals were certified specific-pathogen-free at supply through Charles River Laboratories’ routine SPF/VAF barrier-room health monitoring program, with batch-specific health reports issued for each shipment (publicly accessible at https://www.criver.com/products-services/basic-research/health-reports/europe-asia). Health reports were reviewed on arrival by the attending veterinarian. Animals had not undergone any prior experimental procedures.

Rabbits were housed individually in cages compliant with Directive 2010/63/EU minimum housing requirements (Scanbur, Karlslunde, Denmark). Environmental conditions were controlled at temperature 17 °C–18 °C, humidity 50%–55%, and 12-h light/dark cycle. Animals had free access to standard pelleted rabbit chow (Brogaarden Kaninpiller, Brogaarden A/S, Lynge, Denmark), hay, daily fresh greens, and tap water throughout the study period.

Environmental enrichment was provided in the form of hay, chewing material, wooden sticks, and elevated platforms allowing animals to rest both above and below the structure. Animals were monitored daily by trained personnel for general health, behaviour, food and water intake, and urinary output. Body weight was recorded weekly throughout the study period.

### 2.5 Allocation

Animals were allocated to treatment groups using a weight-stratified allocation approach to ensure comparable baseline weight distributions between groups. Allocation was performed by the principal investigator using a deterministic computer-executed round-robin distribution based on preoperative body weight.

### 2.6 Anaesthesia, analgesia and perioperative care

#### 2.6.1 Anaesthesia

General anaesthesia was induced by subcutaneous administration of fentanyl/fluanisone (Hypnorm®, 0.315 mg/mL fentanyl citrate + 10 mg/mL fluanisone; VetaPharma Ltd, Leeds, United Kingdom) at 0.5 mL/kg, without separate premedication. Animals were maintained under general anaesthesia throughout the procedure, with supplemental oxygen administered via face mask. Where additional anaesthetic depth was required, supplementation was provided with further Hypnorm® and/or sevoflurane. A venous catheter was placed in an ear vein to allow intravenous access. Intraoperative monitoring included pulse oximetry for continuous assessment of heart rate and oxygen saturation. Ocular lubrication (Systane® gel drops) was applied intraoperatively to prevent corneal drying.

#### 2.6.2 Analgesia

Preoperative analgesia was provided using meloxicam (Metacam®) 0.3 mg/kg subcutaneously. Local anaesthesia was administered as a penile nerve block using lidocaine (2 mg/kg) and bupivacaine (1 mg/kg) prior to urethral incision. In addition, topical lidocaine was applied to the surgical field intraoperatively 1-2 times as needed. Postoperative analgesia consisted of meloxicam (0.3 mg/kg subcutaneously twice daily for 5 days). Buprenorphine (Vetergesic®) was administered immediately postoperatively (0.5 mL), followed by 0.3 mL three times daily for up to 4 days, corresponding to approximately 0.01–0.05 mg/kg, based on clinical assessment.

#### 2.6.3 Perioperative and postoperative care

Perioperative antibiotic prophylaxis was administered as a single intramuscular dose of enrofloxacin (Baytril®, 5 mg/kg) immediately prior to surgery. Animals were assessed daily for general condition, surgical site status, and food and water intake. Body weight was recorded at least weekly. Urinary function was closely monitored during the early postoperative period. Absorbent pads were placed in cages to facilitate assessment of urine output. Abdominal palpation was performed at least daily and on clinical indication to detect bladder distension. In cases of suspected urinary retention, ultrasound examination was performed.

### 2.7 Surgical procedure

All surgical procedures were performed by two reconstructive urology surgeons (RH and GT) with extensive clinical experience in urethroplasty. The two surgeons alternated between the roles of primary surgeon and assistant across cases, ensuring that both contributed to all treatment groups and that surgical technique remained consistent throughout the study.

Following urethral catheterisation using a 6 Fr feeding tube (Figure 1A), the penile urethra was exposed through a ventral midline skin incision (Figure 1B). The perineostomy site was opened proximal to the planned urethroplasty (Figure 1C) to prepare for later urinary diversion. The urethra was then carefully mobilised (Figure 1D), and a standardized dorsal urethral defect measuring 10 × 3 mm was created. In scaffold-treated groups, a dorsal onlay graft of identical dimensions was placed on the defect (Figures 1E,F) and sutured with interrupted 6-0 polydioxanone monofilament sutures (PDS II, Ethicon, Somerville, NJ, United States) (Figure 1G). To allow identification of the implantation site at explantation, 6-0 polypropylene monofilament sutures (Surgipro II, Covidien, Mansfield, MA, United States) were placed at the proximal, distal, and lateral margins. In the control group, an identical dorsal urethral defect was created, and the urethral tissue was re-approximated without graft implantation. Corresponding 6-0 polypropylene monofilament sutures (Surgipro II) were placed at the defect margins for identification.

**FIGURE 1:**
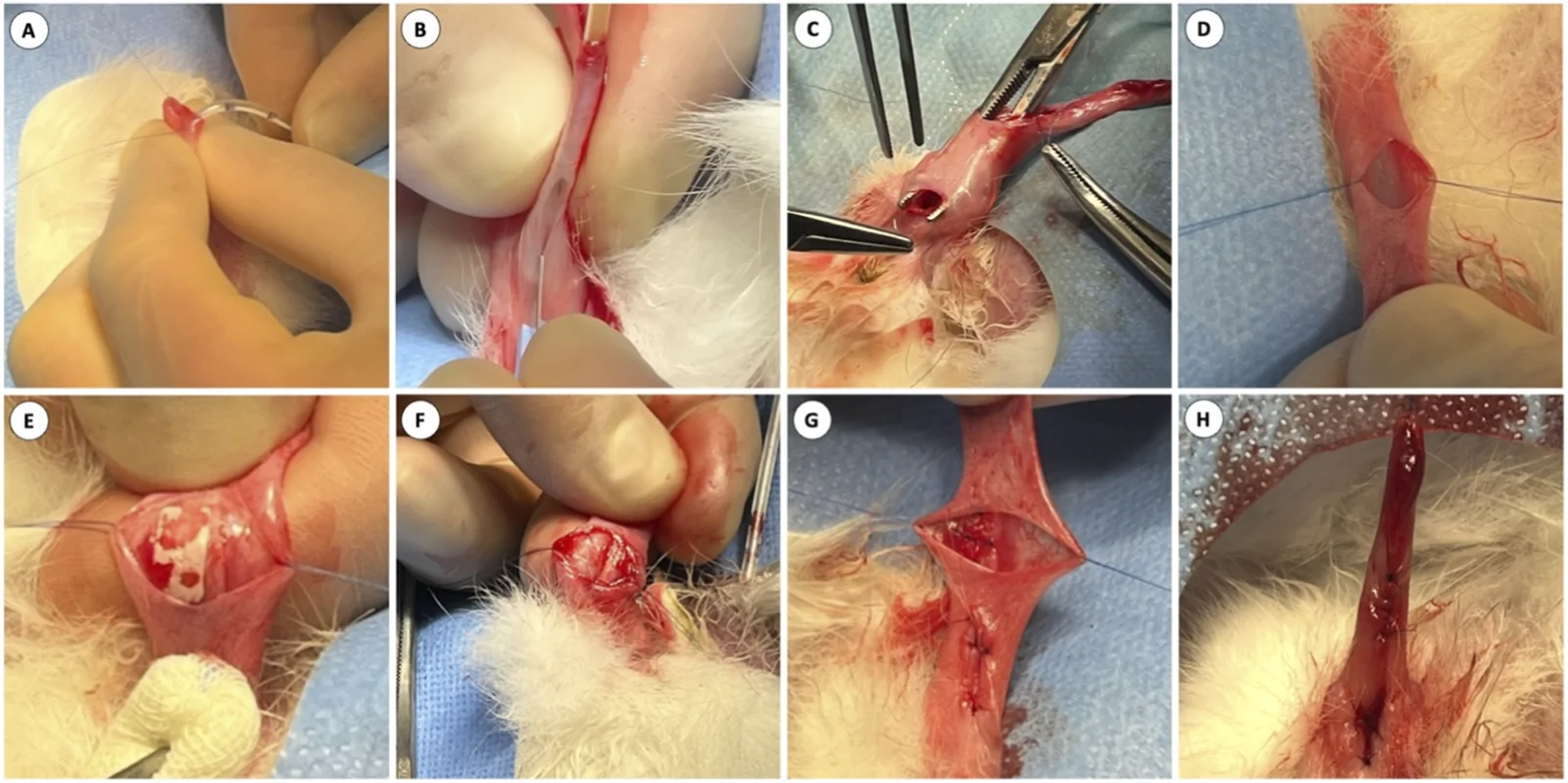
Surgical procedure of dorsal onlay urethroplasty in the rabbit. **(A)** Urethral catheterisation with a 6 Fr feeding tube. **(B)** Ventral midline skin incision to expose the penile urethra. **(C)** Perineostomy site opening for urinary diversion. **(D)** Ventral exposure of the mobilised penile urethra with the 6 Fr feeding tube in place. **(E, F)** Close-up of the scaffold in the dorsal defect prior to suturing. **(G)** Suturing of the scaffold graft to the defect margins with interrupted 6-0 polydioxanone sutures. **(H)** Immediate post-operative view showing standardized surgical result with graft-site sutures and perineostomy for urinary diversion.

Following urethral reconstruction, a perineostomy (≈10 mm) proximal to the urethroplasty site was created by suturing the urethral mucosa to the perineal skin with interrupted 6-0 polydioxanone sutures (PDS II) to divert urine and maintain urethral patency through the follow-up period. The ventral midline skin incision was closed with a 6-0 polydioxanone running suture (PDS II); the standardized post-operative result is shown in Figure 1H. A perineostomy was preferred to an indwelling urethral catheter because rabbits do not reliably tolerate prolonged urethral catheterization, in contrast to the postoperative catheter regimen routinely used in human urethroplasty.

Standardized intraoperative measurements were obtained with the penis held in a stretched position, including distances from the proximal and distal graft margins to the urethral meatus and perineostomy, to enable accurate localization of the implantation site during post-mortem retrograde urethrography.

### 2.8 Outcome assessment

#### 2.8.1 Histological and immunohistochemical analysis

Following euthanasia, urethral specimens were harvested and processed for histological and immunohistochemical analysis. All slides were scanned at ×40 using a Hamamatsu Nanozoomer HT slide scanner. Of the 3-4 cross-sections available per animal, the section showing the largest contiguous expanse of the dorsal urethral plate at the implantation site, with preserved urethral wall architecture and minimal sectioning artefact, was selected by two experienced pathologists (JB, CT) for quantitative analysis. Selection was performed prior to immunohistochemical staining and was blinded to treatment allocation. Sections were reviewed in parallel using hematoxylin-eosin, Masson’s trichrome and CD31-immunohistostained slides cut from sequential levels of the same paraffin block. Analyses were performed within a predefined region of interest (ROI) located at the dorsal aspect of the urethra toward the corpus cavernosum (Figure 2).

**FIGURE 2:**
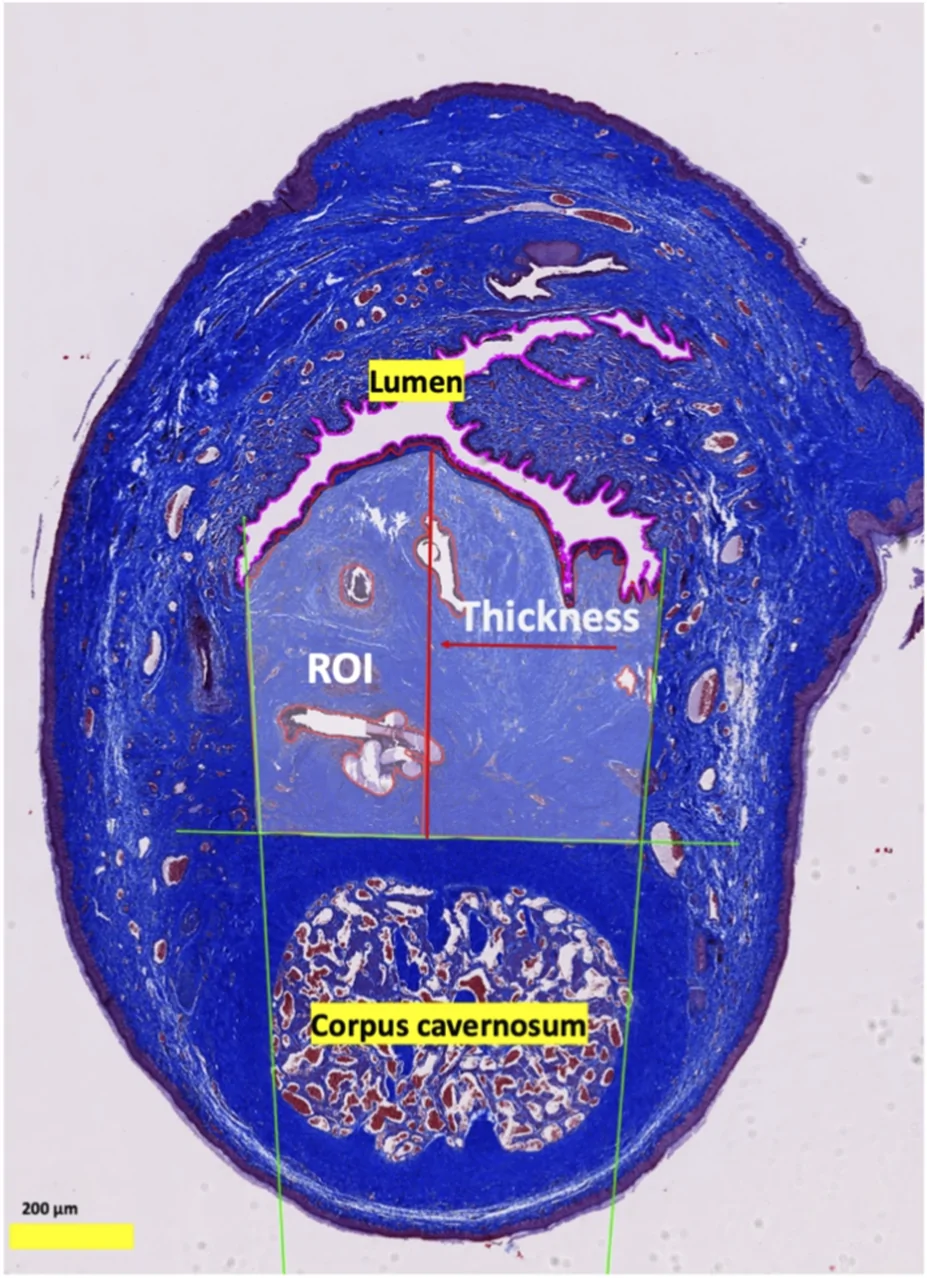
Region of interest (ROI) definition and tissue thickness measurement. Representative Masson’s trichrome-stained cross-section of the rabbit urethra. The predefined ROI (lightly shaded region with green outline) was located at the dorsal urethral wall, extending toward the corpus cavernosum. Tissue thickness (red arrow) was measured as the perpendicular distance from the luminal surface to the outer boundary of the urethral wall. Quantitative analyses of vessel density, fibrosis, and smooth muscle content were performed within the annotated ROI using digital image analysis. Scale bar: 200 µm.

CD31 immunohistochemistry was used to assess vessel density. Vessel density was quantified within the ROI on CD31-stained sections at ×20 magnification. The ROI was subdivided using a standard-sized counting grid overlay of 250 × 250 μm ensuring systematic assessment. CD31-positive vessels were counted in each field and summed across the ROI. A CD31-positive vessel was defined as a discrete endothelial structure with an identifiable lumen and a single layer of flattened endothelial cells, irrespective of erythrocyte content; pre-existing larger muscular vessels and isolated endothelial-cell clusters without identifiable lumen were excluded.

Structures with equivocal luminal architecture were excluded from the count under a pre-agreed 'when in doubt, exclude’ rule; borderline cases were reviewed with a second pathologist (CT) prior to finalization.

All CD31-positive vessels meeting the inclusion criteria were marked with red annotation circles for quantification. Vessel counts were normalized to total ROI area and expressed as vessels per mm^2^. Masson’s trichrome staining was used to assess fibrosis and smooth muscle content. Fibrosis was quantified by digital image analysis (QuPath; v0.5.1, University of Edinburgh, United Kingdom) as the percentage area of collagen within the ROI using a thresholder based on blue channel intensity (Bankhead et al., 2017). The pixel-intensity threshold was calibrated once by a second pathologist (CT) on a training subset of slides, then applied uniformly by the primary analyst (JB) across the cohort without per-slide re-tuning, ensuring between-animal comparability and minimising operator subjectivity.

Smooth muscle content was quantified as the percentage area of smooth muscle within the ROI using a separate Random Forest-based pixel classifier implemented in QuPath, that had been trained by CT.

Re-epithelialization was assessed on hematoxylin-eosin sections within the same predefined ROI used for other histological analyses. Urothelial coverage was quantified as the percentage of the luminal surface across the implantation site demonstrating intact urothelial lining. Missing epithelium was defined as complete or partial denudation. Reactive but continuous urothelium was scored as intact (100%). Re-epithelialization data were available for 23 of 24 animals. One sham animal euthanized at day 13 was excluded from this endpoint analysis, consistent with the exclusion applied to other 12-week outcomes.

Inflammatory cell infiltration was evaluated using a semi-quantitative scoring system (0–4) based on cell density per high-power field in areas of focal inflammation within the ROI. Scores were defined as: 0 = absent (no cells of the relevant lineage in the ROI); 1 = sparse, scattered (1–4/hpf, in areas with inflammation) cells; 2 = moderate (5–10/hpf, in areas with inflammation); 3 = dense, multiple aggregates per high power field or band-like infiltrate; 4 = sheet-like infiltrate occupying a substantial part of the ROI. Lymphocytes, plasma cells, polymorphonuclear cells, macrophages, and giant cells were scored separately. Necrosis was assessed using the same ordinal scale. For each cell type, the ROI was first screened at low magnification (×5–×20) to identify the area of highest cellular density; the corresponding hotspot was then evaluated at ×40 magnification, and a score was assigned based on the cell count per high-power field within that focal area. Scores reflected the maximum focal density rather than the average across the entire ROI, preserving sensitivity to localised inflammatory and foreign-body reactions adjacent to residual scaffold material.

Tissue thickness was defined within the ROI as the perpendicular distance from the luminal surface to the outer boundary of the urethral wall using digital annotation tools and expressed in micrometers (µm).

At the 12-week endpoint, residual scaffold polymer was qualitatively assessed on H&E and Masson’s trichrome sections from all scaffold-treated animals; quantification was not performed. All histological and immunohistochemical assessments were performed by a single pathologist (JB) blinded to treatment allocation. Slides were labeled with blinded specimen identifiers, with the allocation key held by the principal investigator (M.B.M.). Blinding was maintained throughout scoring, including intra-observer re-scoring; group allocation was revealed only after all measurements were finalized. A targeted re-review of specimens showing focal urothelial loss (Section 3.5) was performed by CT, blinded to treatment allocation. To assess intra-observer reliability, six specimens (at least one per treatment group) were randomly selected and re-scored blindly for CD31-positive vessel density by JB at a later time point. Intra-observer agreement was quantified using the intraclass correlation coefficient (ICC(3,1), consistency), interpreted according to Koo and Li (2016).

#### 2.8.2 Urethrography

Retrograde urethrography was performed post-mortem prior to tissue explantation to assess urethral patency. A 6 Fr feeding tube was inserted into a stretched penile urethra and iohexol contrast medium (Omnipaque 240 mg I/mL, GE Healthcare, Chicago, IL, United States) was injected in retrograde fashion. Spot radiographs were acquired during retrograde contrast injection using a mobile C-arm fluoroscopy system (BV Pulsera, Philips Medical Systems, Best, Netherlands), with the animal in the supine position. Urethral patency was assessed qualitatively. Stricture formation was defined as a reduction in urethral diameter of ≥50% at the implantation site compared with the urethral diameter 1 cm distally. Fistula formation was defined by the presence of contrast extravasation or abnormal urethral continuity. Lumen width was quantified on urethrography images using ImageJ (NIH, Bethesda, MD, United States). For each animal, lumen width was measured perpendicular to the urethral axis at two positions: (i) at the implantation site, identified by surgical marker sutures and intraoperative reference measurements, and (ii) at a reference point approximately 1 cm distal to the implantation site. The ratio of these two measurements (implantation-site width/distal reference width) provided a unitless descriptor of luminal calibre, robust to variation in imaging distance, projection angle, and exposure. All urethrographic assessments, both qualitative (patency, stricture, fistula) and quantitative (lumen-width ratio), were performed by the principal investigator on images labeled with study identifiers only; the allocation list was not consulted during scoring, and group allocation was unmasked only after all assessments were complete.

### 2.9 Statistical analyses

Sample size was calculated for the primary endpoint of vessel density. The control value reported by Vaos et al. (2013) [23.33 ± 4.92 (mean ± SD) CD31-positive vessels per ×200 optical field at 84 days following simple urethrotomy in a rabbit urethroplasty model] was used as the reference. Assuming a two-sided alpha of 0.05, a power of 80%, and a between-group mean difference of approximately seven vessels (corresponding to a ∼30% relative increase over the published control value, and within the order of magnitude of inter-graft differences of ∼10–13 vessels reported between distinct urethroplasty grafts in the Vaos series) with a common standard deviation of 5, a two-sample comparison for the primary contrast of CPC versus PLGA indicated that eight animals per group would be required. This per-group size was applied uniformly to all three arms (CPC, PLGA, sham), yielding a total enrolment of 24 animals. The calculation was performed using the ClinCalc online sample-size calculator (Kane SP, ClinCalc.com; accessed 18 June 2024). Note that Vaos et al. (2013) reported vessel density per ×200 optical field, whereas the present study expressed vessel density per mm^2^. This unit difference does not affect the sample-size calculation, which depends only on the ratio of the targeted effect (7 vessels) to the planning standard deviation (5 vessels). This ratio of 1.4 is preserved under any linear unit conversion, and the planned eight animals per group therefore apply equivalently to a per-mm^2^ expression. In the present cohort, the observed within-group standard deviation at the 12-week endpoint was substantially higher than the Vaos reference SD scaled to comparable units, which reduced the statistical power achieved for the primary contrast below the design target (Section 4.2).

Statistical analyses were performed using Stata/MP 18.0 (StataCorp LLC, College Station, TX, United States). Continuous variables were summarized as median [interquartile range], and categorical variables as counts and percentages.

Procedural and safety outcomes were analysed in all animals. Endpoint analyses were performed according to planned follow-up status; animals euthanized before the planned 12-week endpoint were excluded from comparative endpoint analyses.

Overall differences between treatment groups were assessed using Kruskal–Wallis tests for continuous and ordinal variables. Pairwise between-group comparisons (CPC vs. PLGA; CPC vs. sham; PLGA vs. sham) were performed using Wilcoxon rank-sum tests. Pairwise comparisons were not adjusted for multiple comparisons; we report unadjusted p-values for transparency but interpret them as exploratory. Inferential weight is placed on the omnibus Kruskal–Wallis tests and on the primary contrast (CPC versus PLGA on vessel density); pairwise comparisons reported after a non-significant omnibus are interpreted as descriptive directional findings only. Categorical variables were compared using Fisher’s exact test.

Associations between continuous and ordinal histological variables were assessed using Spearman’s rank correlation coefficient. Analyses were performed using complete cases for each outcome. For median between-group differences in vessel density and for Spearman correlations among histological endpoints, 95% confidence intervals were estimated by non-parametric bootstrap with 2,000 resamples and a fixed random seed. A two-sided *p* < 0.05 was considered statistically significant.

## 3 Results

### 3.1 Electrospun scaffolds showed nanofibrous morphology and target thicknesses on SEM

Scanning electron microscopy of representative scaffolds confirmed the fabrication outcome. Surface imaging demonstrated a dense, interconnected nanofibrous architecture characteristic of electrospun matrices (Figure 3A). Cross-sectional examination showed a representative thickness of approximately 300 µm for the monolayer PLGA scaffold (Figure 3B) and approximately 500 µm for the trilayer scaffold (Figure 3C), with discrete layer boundaries within the trilayer not clearly resolvable by SEM. Fiber diameter measurements (n = 50 per scaffold) confirmed a nanoscale morphology for the CPC surface (172 ± 43 nm; Figure 3D) and a microscale morphology for the PLGA monolayer (1,298 ± 187 nm; Figure 3E).

**FIGURE 3:**
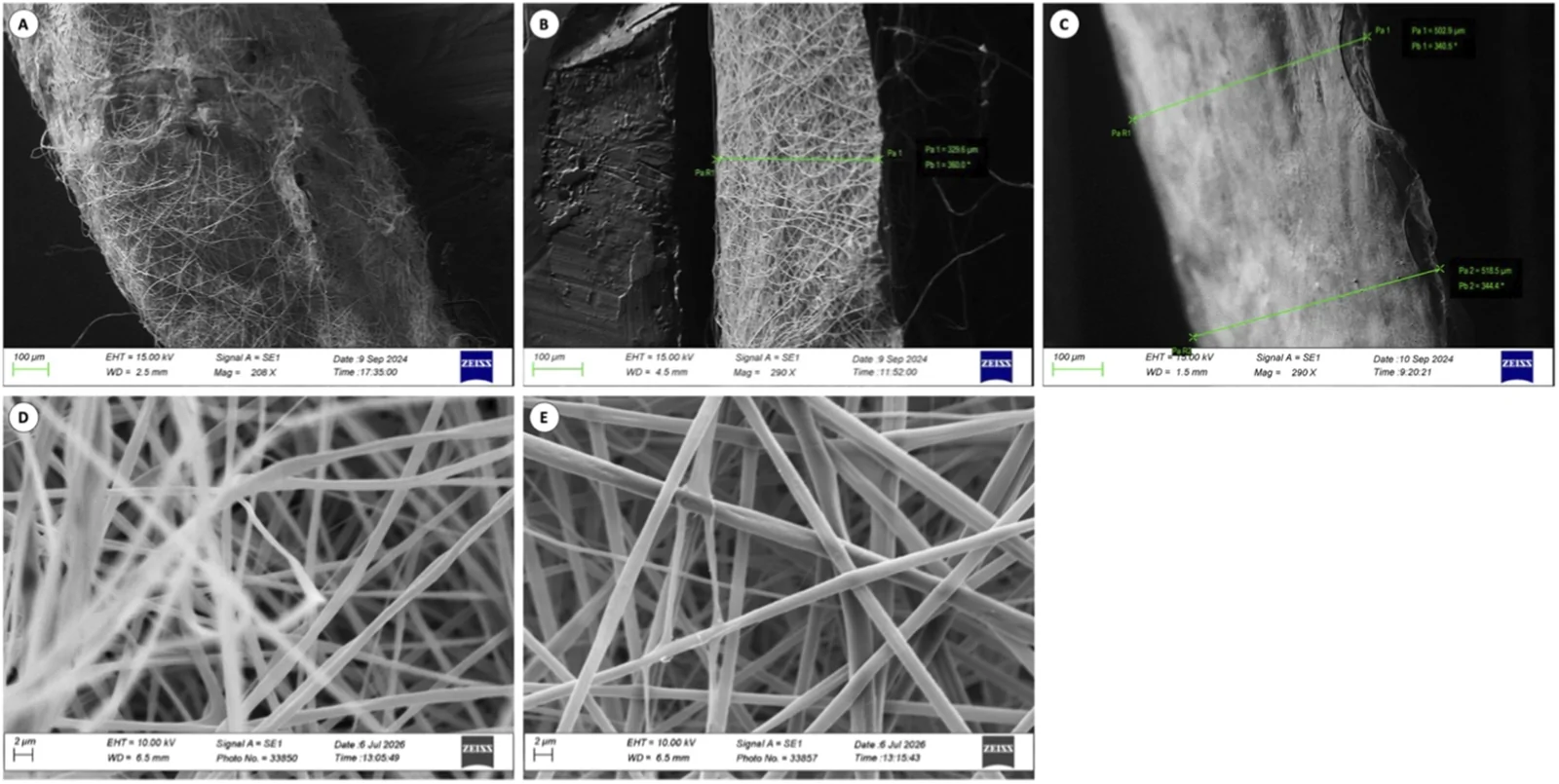
Scanning electron microscopy of fabricated electrospun scaffolds. **(A)** Surface morphology of the trilayer chitosan/PLGA/chitosan scaffold showing dense, interconnected nanofibrous architecture. **(B)** Cross-section of the monolayer PLGA scaffold (∼300 µm). **(C)** Cross-section of the trilayer scaffold (∼500 µm); discrete layer boundaries between the PLGA core and chitosan/PEO outer layers were not distinguishable. **(D)** High-magnification view of the CPC scaffold surface showing individual chitosan/PEO fibers (mean diameter 172 ± 43 nm, n = 50). **(E)** High-magnification view of the PLGA monolayer showing individual PLGA fibers (mean diameter 1,298 ± 187 nm, n = 50). Scale bars: 100 µm **(A–C)**, 2 µm **(D,E)**.

### 3.2 Procedures were completed with comparable recovery across groups

Baseline characteristics, procedural metrics, and weight changes through the 12-week endpoint are summarized in Table 1.

**TABLE 1:** Baseline and procedural characteristics.

Variable	CPC (n = 8)	PLGA (n = 8)	Sham (n = 8)
Baseline weight, g	2,895 [2,745–3,055]	2,945 [2,785–3,195]	2,850 [2,610–2,980]
Procedure time, min	29 [25–32.5]	32.5 [25–46]	26.5 [22–28]
Anaesthesia-related events, n (%)	0 (0%)	3 (37.5%)	1 (12.5%)
Early euthanasia, n (%)	0 (0%)	0 (0%)	1 (12.5%)
Max weight loss, %	−1.79 [−3.58 to 1.24]	−1.13 [−2.65 to 0.89]	0.17 [−3.42 to 3.10]
Net weight change at 12-week follow-up, %	20.96 [18.03–23.98]	20.62 [15.75–21.89]	19.37 [15.36–24.75]

One sham-group animal underwent humane euthanasia at day 13 because of acute-onset bilateral hindlimb paralysis. The clinical presentation was consistent with traumatic lumbar spinal injury, a recognized phenomenon in laboratory rabbits associated with sudden movements within a cage; a definitive cause was not established, but post-mortem examination identified no urethral or perineal lesion attributable to the urethroplasty procedure, and the perioperative course had been unremarkable. The event was therefore judged unrelated to the experimental intervention. Procedural and safety data from this animal were retained, while endpoint analyses were restricted to animals completing the planned 12-week follow-up.

No significant differences were observed between groups in procedure time (p = 0.510), net weight change at 12-week follow-up (p = 0.695), or maximum weight loss (p = 0.728). Intraoperative events occurred in 4/24 animals; all anaesthesia-related. No postoperative complications or infections were observed during follow-up. No macroscopic fistulae, adhesions to surrounding tissues, necrosis, or calculi were observed at 12-week harvest (Supplementary Figure S2). Missing data were limited and occurred primarily in longitudinal weight measurements and procedure time.

### 3.3 Vessel density was lower in scaffold-treated than in sham animals at 12 weeks

Representative CD31-stained sections from each treatment group are shown in Figures 4D–I. Vessel density differed significantly between treatment groups (Kruskal–Wallis p = 0.008; Figure 5). Sham animals exhibited the highest vessel density (median 78.6 [70.1–92.8] vessels/mm^2^), monolayer PLGA the lowest (39.1 [27.6–49.4] vessels/mm^2^), and CPC was intermediate (46.3 [31.9–85.4] vessels/mm^2^).

**FIGURE 4:**
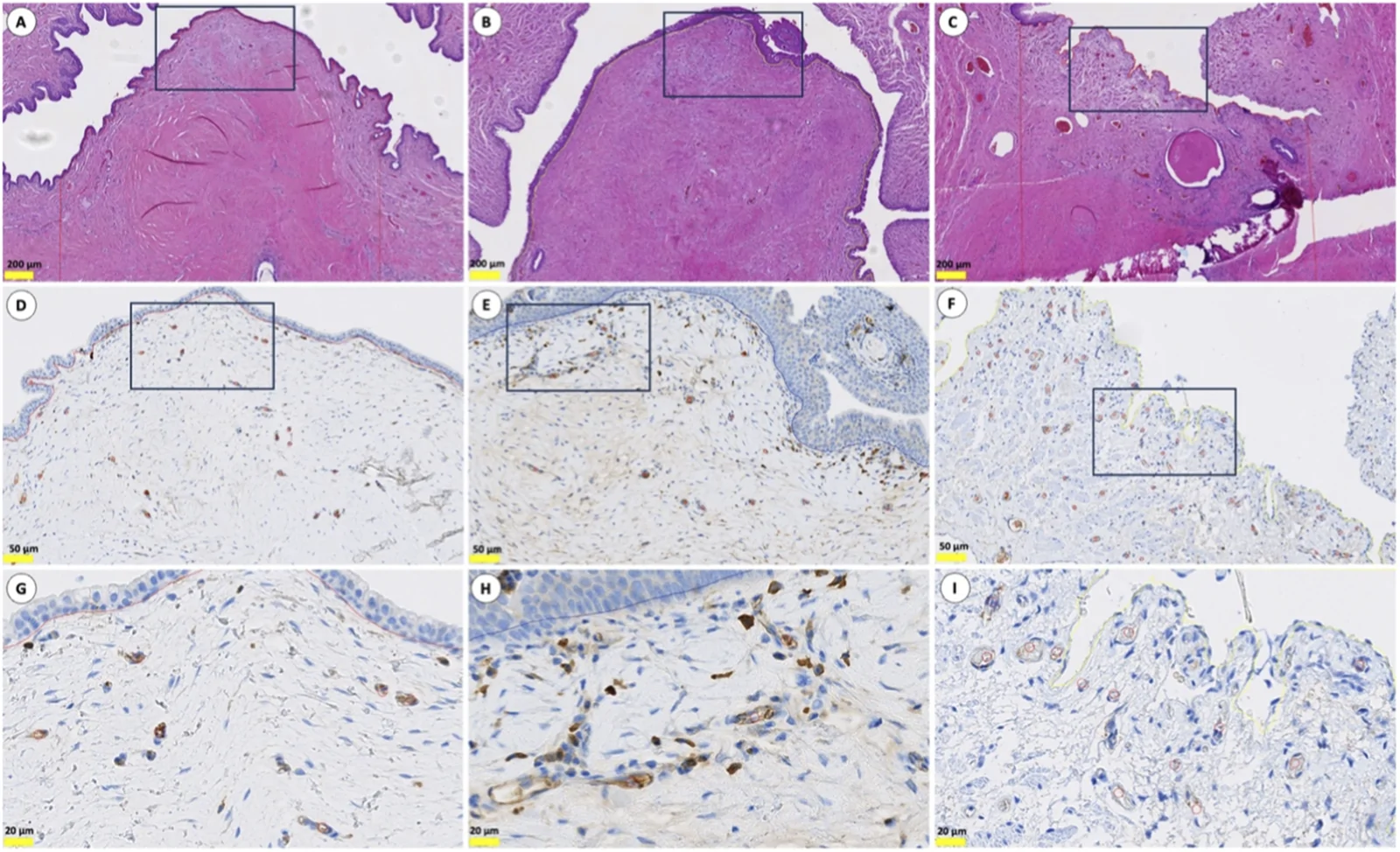
Representative histology of the dorsal urethral plate at 12 weeks. Columns: CPC **(A,D,G)**; PLGA **(B,E,H)**; sham **(C,F,I)**. **(A–C)** Hematoxylin and eosin staining at ×5; black boxes mark the region of interest (ROI) shown at higher magnification in **(D–F)**. **(D–F)** CD31 immunohistochemistry at ×20; black boxes indicate the areas shown at higher magnification in **(G–I)**. **(G–I)** CD31 immunohistochemistry at ×60. Red circles mark CD31-positive vessels counted during quantification. Where visible, yellow outlines indicate the ROI boundary preserved from digital annotation. Brown: CD31 immunoreactivity; blue: hematoxylin counterstain. Scale bars: 200 μm **(A–C)**, 50 μm **(D–F)**, 20 μm **(G–I)**.

**FIGURE 5:**
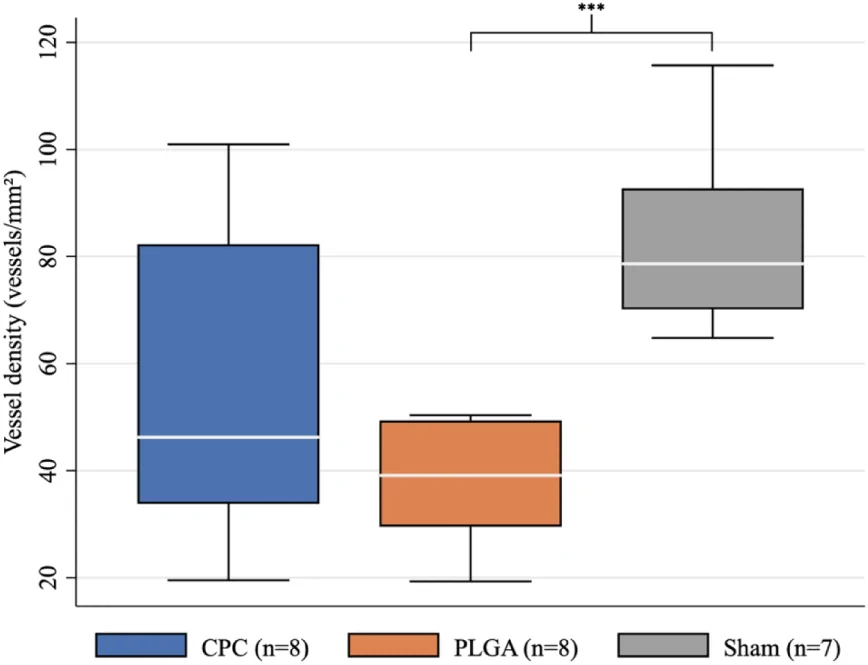
Vessel density by treatment group. Boxplots showing vessel density (vessels/mm^2^) across CPC, PLGA, and sham groups. The central line is the median, boxes the interquartile range, and whiskers the range. Vessel density differed significantly between groups (Kruskal–Wallis p = 0.008). The bracket indicates a significant pairwise comparison by Wilcoxon rank-sum. ***p < 0.001. Non-significant pairwise comparisons are reported in the text.

The CPC-PLGA median difference was 7.2 vessels/mm^2^ (95% bootstrap CI: −27.3 to 41.6); this difference did not reach statistical significance (*p* = 0.248).

In secondary pairwise comparisons, vessel density was significantly higher in sham than in PLGA (median difference 39.5 vessels/mm^2^, 95% bootstrap CI: 19.4 to 59.7; *p* = 0.0008). Vessel density was numerically lower in CPC than in sham (median difference 32.4 vessels/mm^2^, 95% bootstrap CI: −2.7 to 67.4; *p* = 0.074), but the difference did not reach statistical significance. Intra-observer reliability of vessel-density scoring was excellent (ICC(3,1) = 0.97, 95% CI 0.79–1.00).

### 3.4 Scaffold implantation increased tissue thickness without changing fibrosis or smooth muscle content

Tissue thickness differed significantly between treatment groups (Kruskal–Wallis *p* = 0.002; Figure 6A). Representative H&E sections from each group are shown in Figures 4A–C. PLGA-treated animals exhibited the highest tissue thickness (2,446 [2,247–2,536] µm), CPC was intermediate (1,890 [1,566–2,425] µm), and sham animals showed the lowest (1,225 [1,105–1,346] µm).

**FIGURE 6:**
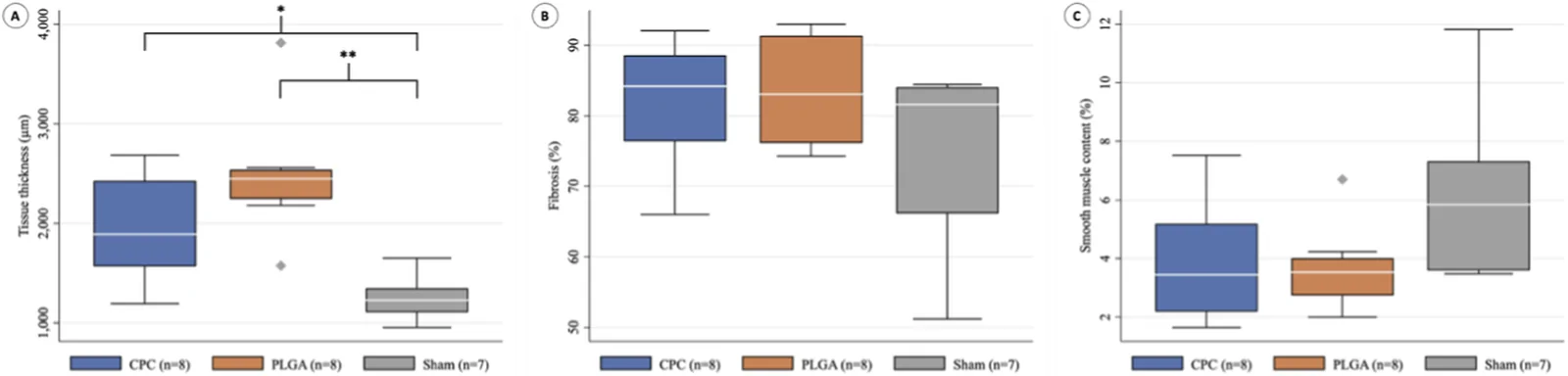
Tissue thickness and remodeling markers by treatment group. **(A)** Tissue thickness (µm), **(B)** fibrosis (%), and **(C)** smooth muscle content (%) across CPC, PLGA, and sham groups. The central line is the median, boxes the interquartile range, and whiskers the range. Tissue thickness differed significantly between groups (Kruskal–Wallis p = 0.002); fibrosis (p = 0.265) and smooth muscle content (p = 0.196) did not. Brackets in panel A indicate significant pairwise comparisons by Wilcoxon rank-sum. *p < 0.05; **p < 0.01.

Pairwise comparisons showed no statistically significant difference between CPC and PLGA (*p* = 0.115). Tissue thickness was significantly higher in CPC compared with sham (*p* = 0.011) and in PLGA compared with sham (*p* = 0.002).

Fibrosis (% area) did not differ significantly between groups (*p* = 0.265), with similar median values across CPC (84.2 [76.4–88.6]%), PLGA (83.1 [76.1–91.4]%), and sham (81.6 [66.2–84.1]%) (Figure 6B).

Smooth muscle content also did not differ significantly between groups (*p* = 0.196), with numerically higher values in sham (CPC 3.4 [2.2–5.2]%, PLGA 3.5 [2.7–4.0]%, sham 5.8 [3.6–7.3]%) (Figure 6C).

### 3.5 Re-epithelialization was high across groups; no ulceration observed

Re-epithelialization did not differ significantly between treatment groups (Kruskal–Wallis p = 0.14). Median urothelial coverage was high across all groups (CPC 100 [97.4–100]%, PLGA 96.5 [95.4–98.9]%, sham 89.7 [31.6–100]%).

Three animals showed focal absence of urothelium: two sham animals (17.3% and 31.6% intact coverage) and one CPC animal (29.4%); the remaining sham animals showed coverage between 88.6% and 100%, and the remaining CPC animals showed coverage ≥96.6%. On blinded pathology review of these three specimens, the denuded regions lacked features of ulceration (no edge reaction, no granulation tissue, and no associated inflammatory infiltrate at the margins of missing urothelium) and were interpreted as denudation of mature urothelium consistent with possible processing artifact rather than failed re-epithelialization. No animal in any treatment group demonstrated ulceration, unresolved wound bed, or tissue necrosis at the 12-week endpoint.

### 3.6 Scaffold implantation was associated with a foreign-body host response

Semi-quantitative inflammatory cell scores by lineage are summarized in Figure 7; full distributions are reported in Supplementary Table S1.

**FIGURE 7:**
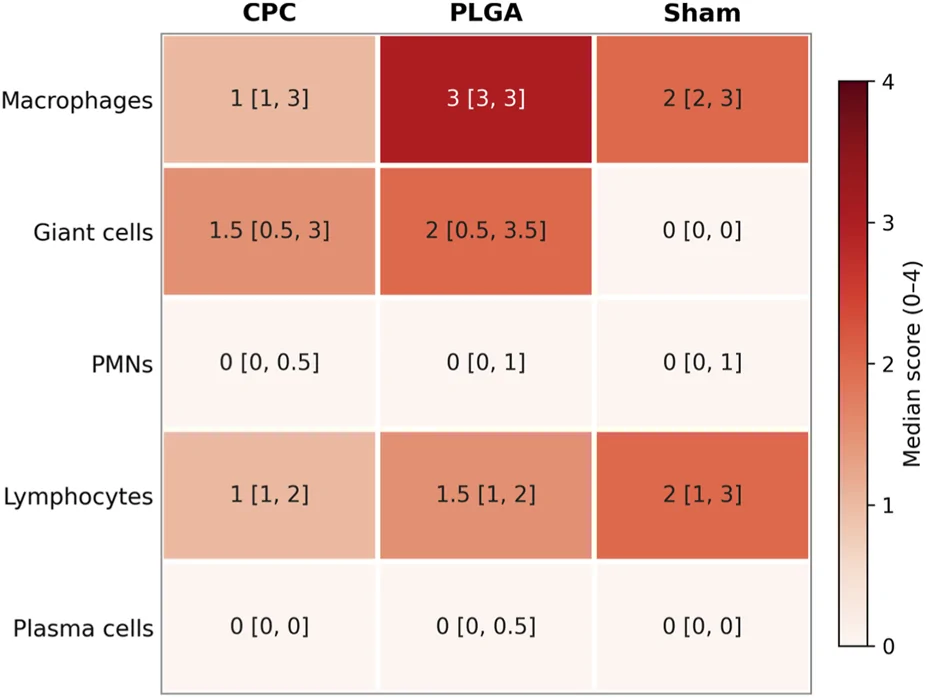
Inflammatory cell scores by treatment group at 12 weeks. Heatmap of median scores (semi-quantitative 0–4 scale) for macrophages, multinucleated giant cells (MNGCs), polymorphonuclear leukocytes (PMNs), lymphocytes, and plasma cells across CPC, PLGA, and sham groups. Cell shading reflects median score (white = 0, dark red = 4); annotations report median [Q1–Q3]. Scaffold-treated animals showed elevated macrophage (p = 0.090) and giant-cell scores (p = 0.026) compared with sham; PMN, lymphocyte, and plasma-cell scores did not differ between groups. Necrosis was absent in all animals (not shown).

Macrophage scores trended toward higher values in scaffold-treated animals (Kruskal–Wallis *p* = 0.090); the highest scores were observed in the PLGA group (PLGA vs. CPC: *p* = 0.062). Giant-cell scores differed significantly between groups (*p* = 0.026): both scaffold groups showed higher infiltration than sham, with no difference between CPC and PLGA (*p* = 0.588). Representative histology of the foreign-body response is shown in Figure 8.

**FIGURE 8:**
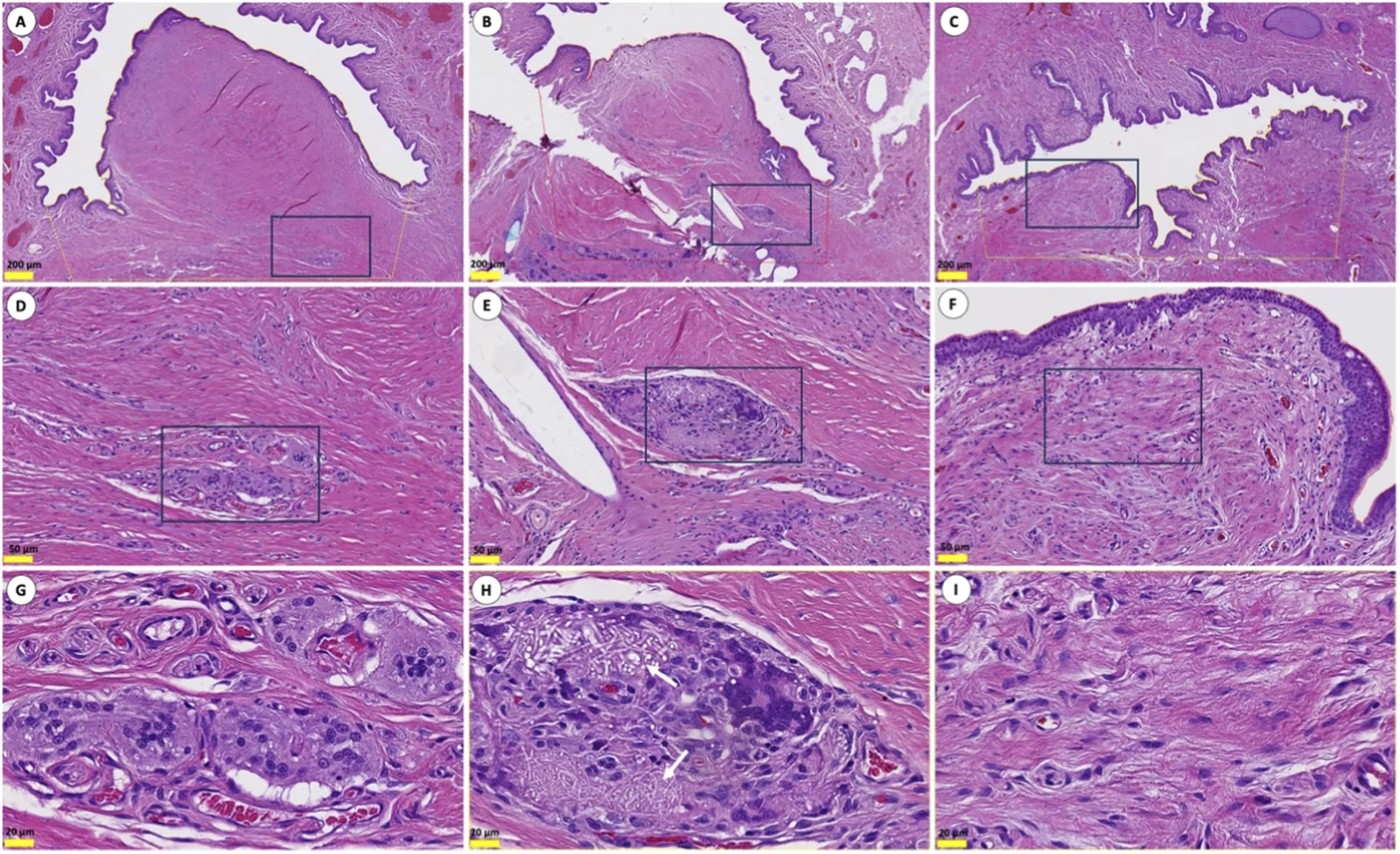
Representative histology of the foreign-body host response at 12 weeks. Columns: CPC **(A,D,G)**; PLGA **(B,E,H)**; sham **(C,F,I)**. **(A–C)** Hematoxylin and eosin staining at ×5; black boxes mark the area shown at higher magnification in **(D–F)**. **(D–F)** H&E at ×20; black boxes indicate the area shown at higher magnification in **(G–I)**. **(G–I)** H&E at ×60. Multinucleated giant-cell aggregates are visible in scaffold-treated animals **(G,H)**; no giant-cell response is present in sham **(I)**. White arrows in **(H)** indicate amorphous material within the aggregate, morphologically consistent with possible scaffold remnants. Scale bars: 200 μm **(A–C)**, 50 μm **(D–F)**, 20 μm **(G–I)**.

Polymorphonuclear-cell scores did not differ between groups (*p* = 0.78). Lymphocyte scores were numerically higher in sham, although the difference did not reach statistical significance (*p* = 0.51); plasma-cell scores did not differ between groups (*p* = 0.12). Necrosis was absent in all animals.

At the 12-week endpoint, identifiable scaffold polymer was observed only rarely within sections from scaffold-treated animals. Isolated foci of amorphous material adjacent to or within foreign-body giant-cell aggregates were occasionally observed within the ROI (Supplementary Figure S1; also visible in Figure 8H); on pathology review the majority of these foci were considered most consistent with remnants of the non-absorbable polypropylene marker sutures placed at the defect margins (Section 2.7), with confirmed residual scaffold polymer identified only rarely. The histological scarcity of identifiable scaffold material at 12 weeks does not by itself establish the extent of *in vivo* scaffold degradation, since residual PLGA-based material can be difficult to distinguish from surrounding connective tissue or suture remnant on H&E alone. Quantification of residual scaffold material was therefore not undertaken (Section 4.7).

### 3.7 Vascularization was inversely associated with fibrosis and tissue thickness

Vessel density was inversely correlated with tissue thickness (Spearman’s ρ = −0.59, 95% bootstrap CI: −0.88 to −0.30; *p* = 0.004) and fibrosis (ρ = −0.61, 95% bootstrap CI: −0.93 to −0.29; *p* = 0.003), while tissue thickness was positively correlated with fibrosis (ρ = 0.48, 95% bootstrap CI: 0.09 to 0.86; *p* = 0.022). No statistically significant correlations were observed between vessel density and macrophage score (ρ = −0.38, *p* = 0.075) or smooth muscle content (ρ = 0.30, *p* = 0.167).

### 3.8 Urethral patency was preserved in all groups at 12 weeks

Retrograde urethrography demonstrated radiographic patency in all 23 animals completing the planned 12-week endpoint (Figures 9A–C), with no fistulae or contrast extravasation observed.

**FIGURE 9:**
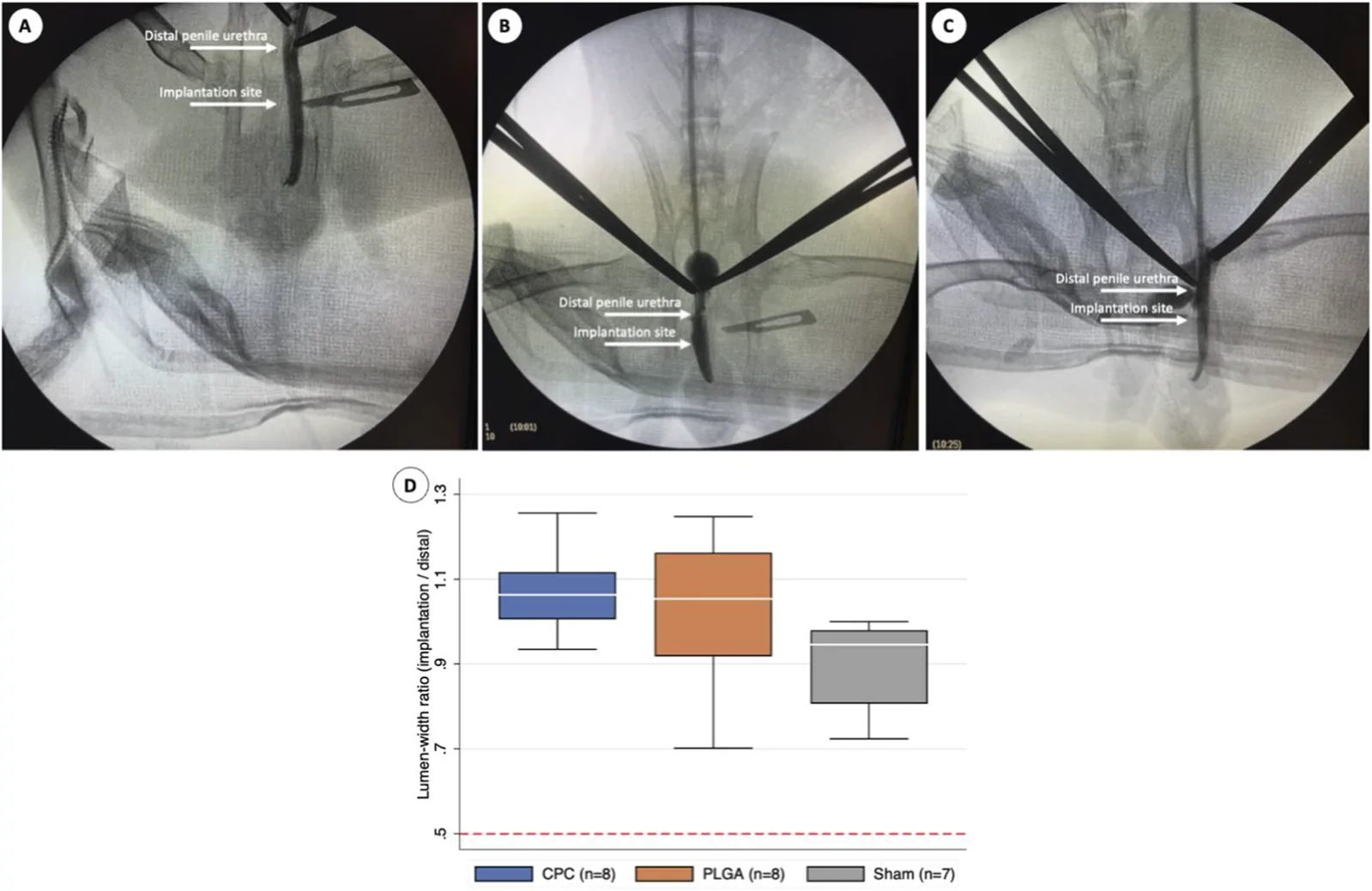
Representative retrograde urethrograms at 12 weeks post-implantation. **(A)** CPC; **(B)** PLGA; **(C)** sham. Upper arrow: distal penile urethra; lower arrow: implantation site. The contrast column is patent throughout in all three groups. **(D)** Lumen-width ratios (implantation/distal reference) per animal by treatment group. Central line: median; box: interquartile range; whiskers: range. Dashed red line indicates the prespecified ≥50% stricture criterion (ratio ≤ 0.50); no animal met this criterion. CPC 1.06 [1.01–1.12]; PLGA 1.05 [0.92–1.16]; sham 0.95 [0.81–0.98]. Kruskal–Wallis p = 0.06.

Quantitative analysis of lumen width at the implantation site relative to the urethra 1 cm distally (Figure 9D) confirmed that no animal met the predefined ≥50% stricture criterion (lowest observed ratio 0.70). Between-group differences in continuous lumen width ratios did not reach statistical significance (CPC 1.06 [1.01–1.12]; PLGA 1.05 [0.92–1.16]; sham 0.95 [0.81–0.98]; Kruskal–Wallis *p* = 0.06). Radiographic assessment of perineostomy status at the 12-week endpoint showed spontaneous closure in 10/23 animals (CPC 4/8, PLGA 2/8, sham 4/7), without significant between-group difference (Fisher’s exact *p* = 0.574).

## 4 Discussion

### 4.1 Principal findings

In this study, we report the first controlled *in vivo* evaluation of a trilayer chitosan/poly(L-lactide-co-glycolide)/chitosan composite electrospun scaffold (CPC) in a rabbit dorsal onlay urethroplasty model, compared against a monolayer PLGA scaffold and a non-scaffolded surgical sham control. Both scaffolds were technically feasible to implant and were well tolerated throughout the 12-week follow-up. Contrary to our primary hypothesis, vessel density did not differ significantly between CPC and PLGA at the 12-week endpoint (*p* = 0.248), and the overall between-group difference (*p* = 0.008) was driven by higher values in sham animals than in PLGA-treated animals (*p* = 0.0008). Macrophage scores trended lower in CPC than in PLGA (*p* = 0.062), and multinucleated giant-cell scores were significantly higher in both scaffold groups than in sham (*p* = 0.026), consistent with a foreign-body host response. Scaffold-treated animals showed greater tissue thickness at the implantation site than sham (*p* = 0.002). Vessel density was inversely correlated with both tissue thickness (Spearman *ρ* = −0.59, *p* = 0.004) and fibrosis (*ρ* = −0.61, *p* = 0.003), defining a coherent remodeling signature in which scaffold-driven repair produced thicker, less-vascularized tissue. Retrograde urethrography demonstrated preserved patency in all 23 animals completing follow-up, with no animal meeting the prespecified ≥50% stricture criterion.

### 4.2 Vascularization at 12 weeks: temporal context

The direction of effect (higher vessel density in CPC than in PLGA) matched the *in vitro* pro-angiogenic activity reported for chitosan-containing constructs (Huang et al., 2020; Abbas et al., 2024). The observed CPC-PLGA median difference (Δ = 7.2 vessels/mm^2^) closely matched the prespecified clinically meaningful difference of seven used in the sample-size calculation; however, within-group variability exceeded the planning assumption (assumed SD = 5), reducing power below the design target. With eight animals per group, this increased the minimum detectable effect to approximately 34 vessels/mm^2^ at 80% power (α = 0.05) and widened the 95% bootstrap CI around the CPC-PLGA difference to −27.3 to 41.6 vessels/mm^2^. Due to the absence of a standard *post hoc* procedure for the rank-based primary analysis, these values were approximated using a two-sample t-test framework based on the observed pooled standard deviation. A definitive demonstration of CPC superiority on vascularization would require earlier sampling, a larger sample, or both.

The pro-angiogenic effect of chitosan is most pronounced during the early host response, when chitosan oligosaccharides act on macrophage pattern-recognition receptors to drive M2 polarization and downstream secretion of VEGF, epidermal growth factor (EGF), and insulin-like growth factor-binding protein 5 (IGFBP-5) (Huang et al., 2020). In a rabbit dorsal urethral defect model, Jin et al. (2025) recently showed that a multilayered hydrogel scaffold combining poly(vinyl alcohol), carboxymethyl chitosan, and adipose-derived stem cells regulates macrophage polarization to accelerate the inflammation-to-proliferation transition and promote scar-free urethral healing. By 12 weeks, both the early angiogenic peak and the bulk of chitosan-layer resorption are likely to have occurred, leaving a remodeling field dominated by the residual PLGA core and its associated foreign-body response. The 12-week endpoint was prespecified to align with the timepoint at which comparable rabbit urethroplasty studies characterize established rather than forming vascular networks (Vaos et al., 2013; Chung et al., 2014), and is consistent with the slower hydrolytic degradation expected of the high-lactide (85:15) PLGA core relative to lower-lactide compositions (Makadia and Siegel, 2011). This design choice constitutes an explicit limitation with respect to detecting the acute chitosan-driven angiogenic peak, which occurs earlier.

The pro-angiogenic and biocompatible properties previously demonstrated for a chitosan/poly(L-lactide)/chitosan trilayer construct (Abbas et al., 2024) translated to only a modest directional trend in the present *in vivo* evaluation. We substituted PLGA (Resomer LG 857 S, 85:15, ester-terminated) for PLA in the core layer to combine controllable hydrolytic degradation with the broad regulatory acceptance of PLGA in medical-device applications. 85:15 ester-terminated PLGA has a slow *in vivo* degradation profile, with mechanical integrity and mass typically retained for several months in published *in vitro* and *in vivo* models (Makadia and Siegel, 2011). Identifiable residual scaffold polymer was nevertheless rarely seen on H&E at the 12-week endpoint in the present cohort (Section 3.6); whether this reflects partial *in vivo* degradation, integration with regenerating tissue, or limited histological distinguishability from surrounding connective tissue on H&E alone cannot be determined from the present data, and motivates inclusion of construct-specific *in vitro* degradation characterization and polymer-specific staining in future studies (Section 4.7).

This in vitro-to-in vivo discrepancy likely reflects host healing kinetics and the rabbit’s intrinsic urethral healing capacity, which together attenuate inter-material differences at intermediate timepoints. The present data therefore refine rather than reject the hypothesis that adding chitosan layers atop a synthetic polyester core confers a measurable *in vivo* advantage.

A further consideration is that the CPC and PLGA scaffolds differ not only in the surface chemistry but also in the architecture. The trilayer construct is thicker (∼500 µm versus ∼300 µm) and its chitosan surface layers exhibit fiber diameters an order of magnitude smaller than the PLGA monolayer (172 ± 43 nm versus 1,298 ± 187 nm). Fiber diameter influences protein adsorption, cellular attachment, and infiltration, and chitosan additionally exhibits hydrophilic swelling in aqueous environments, unlike PLGA (Dash et al., 2011; Abbas et al., 2024). The observed biological differences between the two scaffolds therefore reflect the combined influence of composition, architecture, and swelling behaviour, and cannot be attributed to chitosan chemistry alone. Disentangling these contributions would require architecturally matched controls with equivalent thickness and fiber diameter.

The directional finding that vessel density in scaffold-treated animals was lower than in sham at 12 weeks warrants explicit comment. Two factors plausibly contribute. First, the rabbit urethra revascularizes robustly after primary closure of a small dorsal defect, and the sham group represents an upper bound for vascularization in this model rather than a vascularization-deficient comparator. Second, scaffold-driven tissue regeneration produced thicker tissue at the implantation site (Section 4.4) over which a comparable absolute vessel population was distributed, biasing the per-area metric downward; this is consistent with the inverse vessel-thickness correlation reported in Section 3.7. The case for use of CPC or monolayer PLGA in this model at 12 weeks therefore rests on the scaffold’s structural and substrate-providing role rather than on enhanced angiogenesis, and is supported by the universal urethral patency and absence of ulceration observed across all groups (Sections 4.5, 4.6).

### 4.3 Foreign-body response and chitosan-mediated macrophage modulation

Scaffold implantation elicited a foreign-body response in both treatment groups. Macrophage and multinucleated giant-cell infiltration was higher in scaffold-treated animals than in sham, with significantly elevated giant-cell scores (*p* = 0.026) and a non-significant trend toward higher macrophage scores (*p* = 0.090). The macrophage comparison provided the most defensible signal in favour of the trilayer design: median macrophage scores were lower in CPC than in PLGA (1 vs. 3; *p* = 0.062). Although this difference did not reach statistical significance, it is consistent with chitosan attenuating the foreign-body response, possibly through its established capacity to favour M2 over M1 macrophage polarization via chitosan-oligosaccharide signaling (Huang et al., 2020). PLGA implants drive a stereotyped macrophage and foreign-body giant-cell signature partly through acidic by-products of polymer hydrolysis (Ji et al., 2012); chitosan may dampen this signal through its reactive functional-group chemistry that supports favourable cell-material interactions and through oligosaccharide-mediated immunomodulation of macrophages (Huang et al., 2020; Abbas et al., 2024).

The chitosan-mediated modulation proposed here is inferred from semi-quantitative macrophage counts rather than direct phenotypic markers; the lower macrophage scores in CPC therefore cannot be interpreted as direct evidence of M2 skewing. Because multinucleated giant cells form by macrophage fusion, their phenotype reflects the polarization of the underlying macrophage population. We did not perform immunohistochemistry for canonical M1/M2 polarization markers (e.g., CD86/iNOS versus CD206/Arg-1) on either the macrophage population or the multinucleated giant-cell aggregates. Contemporary work has shown that biomaterial-induced giant cells express both pro- and anti-inflammatory markers in comparable abundance and may best be classified as M1-multinucleated giant cell (MNGC) or M2-MNGC according to the polarization of their macrophage precursors (Barbeck et al., 2016; Miron and Bosshardt, 2018). The elevated giant-cell scores in both scaffold groups therefore do not necessarily indicate a uniformly pro-inflammatory population, and the absence of phenotyping is a meaningful limitation when interpreting the chitosan-modulation hypothesis. In human bone biopsies, synthetic and xenogeneic biomaterials elicit distinct macrophage polarization profiles (Zhang et al., 2019), underscoring that count alone is an insufficient readout of foreign-body response quality.

### 4.4 A coherent host response signature: vessel-thickness-fibrosis coupling

The inverse correlations between vessel density and both tissue thickness (Spearman *ρ* = −0.59) and fibrosis (*ρ* = −0.61), together with the positive correlation between thickness and fibrosis (*ρ* = 0.48), describe a coherent axis of host response in which scaffold-supported remodeling produced thicker, more fibrotic, and less vascularized tissue. These correlation analyses were not prespecified as confirmatory endpoints and should be interpreted as exploratory and hypothesis-generating, given the sample size and the number of comparisons undertaken. This pattern is consistent with the behaviour of synthetic scaffolds at intermediate timepoints, where sustained foreign-body response shifts the balance from constructive vascularization toward fibrotic encapsulation (Dickenson et al., 2026), and where sustained M2 macrophage polarization supports ongoing vascularization in tissue-engineered constructs (Zhao et al., 2022).

In our cohort, sham animals showed median fibrosis of 81.6%, only marginally lower than the 83%–84% observed in scaffold-treated groups, with no statistically significant between-group difference (Kruskal–Wallis *p* = 0.265). This near-equivalence in the absence of any scaffold material suggests that the dominant contributor to the measured collagen signal is native urethral and adjacent corpus cavernosum collagen captured within the ROI, rather than scaffold-induced scar formation, and limits the discriminative value of fibrosis as a between-group endpoint in this anatomical region. Beyond the anatomical confound, the dominant driver of elevated fibrosis across all groups may be the surgical injury itself rather than the implanted material, a contribution this design cannot disentangle. The 12-week endpoint captures established rather than evolving remodeling, and although serial sampling at earlier timepoints could characterize the kinetics of fibrotic deposition, it would not necessarily separate scaffold-driven from operation-driven contributions. Future studies could improve resolution by restricting the ROI to the urethral wall proper, co-registering fibrosis quantification with smooth-muscle staining to exclude native collagen-rich structures, or benchmarking against the contralateral uninjured urethra to isolate the material-specific component.

### 4.5 Re-epithelialization was complete across groups, without ulceration features

Quantification of urothelial coverage at 12 weeks demonstrated essentially complete re-epithelialization across treatment groups (CPC median 100% [97.4–100]; PLGA 96.5% [95.4–98.9]; sham 89.7% [31.6–100]), with no statistically significant between-group difference (Kruskal–Wallis *p* = 0.14). Three specimens showed focal absence of urothelium: two sham animals (17.3% and 31.6% intact coverage) and one CPC animal (29.4%). On blinded pathology review, these denuded regions consistently lacked features of true ulceration: no edge reaction, no granulation tissue, and no associated inflammatory infiltrate were observed at the margins of the missing epithelium in any of the three specimens. The pathology read was therefore consistent with denudation of mature urothelium rather than failed biological re-epithelialization, and a contribution of mechanical or processing-related artifact cannot be excluded.

Despite the focal denudation observed in three specimens, no animal demonstrated ulceration or unresolved wound bed (Section 3.5). The biological signal from this readout is therefore that dorsal onlay urethroplasty, whether performed with the trilayer CPC scaffold, monolayer PLGA, or primary closure, supports complete and quiescent re-epithelialization in this model. Implantation of the scaffold did not obstruct urothelial coverage over the dorsal urethral plate, and the absence of ulceration in scaffold-implanted animals provides direct histological evidence of biocompatibility at the luminal interface. The rapid intrinsic re-epithelialization capacity of the rabbit urethra is illustrated by the sham animal euthanized at day 13, which already demonstrated 94% urothelial coverage at that early timepoint, consistent with the well-documented healing reserve of the rabbit urethra (Abbas et al., 2023) (Section 4.7).

### 4.6 Urethral patency was preserved across all groups

Although the primary endpoint did not establish CPC superiority over PLGA on vascularization, both scaffolds maintained urethral patency throughout follow-up. Retrograde urethrography demonstrated preserved patency in all 23 animals completing the 12-week endpoint, with no animal meeting the prespecified ≥50% stricture criterion. Lumen-width ratios were numerically wider in scaffold groups (CPC 1.06 [1.01–1.12]; PLGA 1.05 [0.92–1.16]) than in sham (0.95 [0.81–0.98]); the overall between-group difference approached but did not reach statistical significance (Kruskal–Wallis *p* = 0.06). The directional pattern is consistent with the structural role of an onlay graft. Urethral strictures result principally from spongiosal fibrosis during healing (Hampson et al., 2014), and a synthetic scaffold can provide a template for lumen-supporting tissue regeneration during this process (Wan et al., 2020). However, the rabbit’s intrinsic urethral healing capacity is sufficient to prevent stricture in most unsupported defects in this model (Chung et al., 2014; Abbas et al., 2023), and a definitive demonstration of scaffold-mediated stricture protection would require a larger defect, a larger sample size, or longer follow-up. Clinically, buccal mucosa graft urethroplasty in humans is associated with a re-stricture rate of approximately 19% at 45 months (Spilotros et al., 2017), and any synthetic alternative will need to be evaluated against this benchmark.

The perineostomy used for urinary diversion provided protective separation of urine flow from the urethroplasty site during the critical early healing phase and may have contributed to the absence of postoperative urinary leakage, urinoma, or wound infection observed across all groups. This diversion strategy is broadly analogous to the post-operative urethral catheterization used in human urethroplasty. Spontaneous perineostomy closure occurred in approximately 43% of animals (10/23) without significant between-group difference (Fisher’s exact *p* = 0.574), providing useful baseline data for future rabbit urethroplasty studies using perineostomy diversion.

Beyond adult urethral stricture, the biological criteria validated in this cohort, namely, complete re-epithelialization without ulceration, preserved patency, and a controlled foreign-body response, are also of considerable interest in hypospadias surgery, where tissue-engineered substitutes have been proposed to address the donor-tissue limitations of autologous preputial skin or buccal mucosa (Tsachouridis et al., 2023). The rabbit dorsal onlay model employed here has been characterized as relevant to preclinical hypospadias research (Abbas et al., 2023), supporting the broader applicability of the present host-response and re-epithelialization findings to hypospadias-directed scaffold development. Pediatric clinical translation would nevertheless benefit from evaluation in a developmentally and anatomically representative model, in which the ventrally deficient urethral plate characteristic of hypospadias is recapitulated.

### 4.7 Strengths, limitations and translational considerations

The strengths of this study are its controlled three-arm design with a sham comparator, weight-stratified allocation across two enrolment periods, blinded outcome assessment, use of a prespecified primary endpoint with an *a priori* sample-size calculation, and breadth of histological, immunohistochemical, and radiographic outcomes assessed in a single cohort. All surgical procedures were performed by two highly experienced reconstructive urology surgeons at the only Danish centre performing adult urethroplasty for stricture disease. The study extends the trilayer composite concept previously characterized *in vitro* (Abbas et al., 2024) into a rigorously controlled *in vivo* evaluation in a clinically relevant model. The inverse vessel-thickness-fibrosis correlations reported here quantitatively describe a host-response signature that has been described qualitatively in earlier urethral tissue-engineering literature (Ławkowska et al., 2023).

Several limitations should be considered when interpreting the findings. The most consequential is species: the rabbit corpus spongiosum has an excellent collateral blood supply that gives the rabbit urethra superior intrinsic healing capacity compared with the human urethra, a recognized characteristic of the rabbit urethroplasty model that may attenuate apparent differences between scaffold and sham conditions that would manifest more strongly in clinical practice (Abbas et al., 2023). Sample size was determined by *a priori* power calculation and constrained in accordance with 3R principles; while sufficient to detect approximately one-standard-deviation between-group differences, this design was underpowered to resolve smaller inter-scaffold effects. No unsutured-defect control was included on ethical grounds, as unapproximated urethral defects may cause urinary extravasation and elevated morbidity; consequently, the scaffold-versus-sham contrast reflects combined material and suture-tension effects, while the CPC-versus-PLGA contrast isolates material-specific effects under identical suture conditions. The single 12-week endpoint provides a snapshot of established remodeling but does not capture the early angiogenic and immunomodulatory window during which chitosan-mediated effects are most prominent in published *in vivo* urethral work (Jin et al., 2025); resolving the kinetics of the chitosan contribution would require either larger cohorts or serial-sampling designs that necessarily increase animal use, a trade-off future work will need to balance against 3R obligations. Histological analyses were performed by a single pathologist blinded to allocation; intra-observer reliability of the primary endpoint was quantified (ICC(3,1) = 0.97; Section 3.3). Supplementary immunofluorescence validation of vessel density was not performed. Functional evaluation was limited to radiographic patency and lumen-width ratio; biomechanical testing of explanted urethras (compliance, burst pressure) and *in vivo* urodynamic assessment (uroflowmetry, cystometry) were not performed, and are required to establish functional equivalence to native urethra or to buccal mucosa graft repair. The multinucleated giant-cell population was not phenotyped, leaving open the question whether the elevated giant-cell counts in scaffold-treated groups represent an M2-leaning, regeneration-supportive infiltrate or a more pro-inflammatory M1-dominated population (Barbeck et al., 2016; Miron and Bosshardt, 2018). Scaffold thickness was characterised by SEM on representative samples rather than on individual implants, because SEM sample preparation is destructive; consistency across the implanted cohort therefore rested on standardised electrospinning duration rather than on per-construct measurement. Gas chromatographic quantification of residual solvent was not performed; the absence of local toxicity or abnormal inflammatory response across the *in vivo* cohort supports the biocompatibility of the post-processing protocol (Section 2.2). Residual scaffold material could not be quantified histologically, and *in vivo* degradation kinetics of the constructs were not characterized; *in vitro* degradation profiling matched to the implantation timeframe, or polymer-specific staining, would strengthen interpretation of the residual-material findings. The dorsal aspect ROI used for quantitative analysis captures collagen-rich tissue adjacent to the corpus cavernosum, limiting the discriminative value of fibrosis as a between-group endpoint. Focal absence of urothelium was observed in three specimens across treatment groups; on blinded pathology review these regions lacked ulceration features and were interpreted as denudation rather than failed re-epithelialization (Section 4.5). Retrospective haematoxylin and eosin review cannot reliably distinguish *in vivo* denudation, for example, from low-grade mechanical trauma during the postoperative period, from peri- and post-mortem urothelial loss occurring during terminal urethrography, specimen harvesting, embedding, sectioning, or staining, and an artefactual contribution to the apparent variability in re-epithelialization cannot be excluded.

Terminal retrograde urethrography was constrained by equipment, animal positioning, and the perineostomy, which limited the achievable projection angles. Antegrade urethrography was not performed: the perineostomy did not provide reliable antegrade access, and post-mortem access was precluded by the specimen preparation workflow.

The variable maintenance of perineostomy diversion across the cohort (Section 4.6) produced differential exposure of the urethroplasty site to urine: animals whose perineostomy closed during follow-up resumed voiding through the urethroplasty site at variable timing, exposing regenerating tissue to urine flow, urinary salts, and shear stress, while animals with persistently open perineostomies maintained urinary diversion throughout. Urine exposure is an established modulator of urethral wound healing and inflammatory tone (Hampson et al., 2014; Jin et al., 2025), and this differential exposure represents a source of within-group biological variability that treatment allocation alone does not capture.

Beyond the specific construct evaluated, the rabbit dorsal onlay urethroplasty model employed here demonstrated an excellent safety and tolerability profile, with no procedure-related mortality, no perineostomy-related complications, no postoperative infections, no ulceration of the urethral plate on histological review, and 100% radiographic patency at 12 weeks across all groups. This baseline performance supports the model’s utility as a reproducible platform for controlled comparison of subsequent urethral scaffolds and time-course studies.

Although the present study focused on dorsal onlay urethroplasty for acquired urethral defects, the findings also have potential relevance for congenital urethral reconstruction, particularly hypospadias repair. Complex and reoperative hypospadias cases often require tissue substitution in an environment characterized by compromised vascularity, fibrosis, and limited availability of local tissue, challenges that parallel those encountered in urethral stricture surgery. The ability of the CPC scaffold to support re-epithelialization, maintain luminal patency, and integrate without procedure-related complications suggests that composite electrospun scaffolds may ultimately have utility as adjuncts or alternatives to autologous grafts in staged hypospadias reconstruction. In particular, the directional pattern of lower macrophage scores observed with the chitosan-containing construct may be relevant in hypospadias surgery, where excessive inflammation and fibrosis contribute to fistula formation and repair failure. While extrapolation from an adult rabbit urethroplasty model to pediatric congenital reconstruction requires caution, the present data support further investigation of chitosan-modified composite scaffolds in preclinical models specifically designed to address the reconstructive demands of hypospadias surgery.

### 4.8 Future directions

Several directions for further work follow logically. Earlier endpoints, at 2 and 4 weeks post-implantation, would directly characterize the chitosan-mediated angiogenic and immunomodulatory window in this model and could be paired with M1/M2 macrophage and giant-cell phenotyping by immunohistochemistry to test the polarization hypothesis. Mechanical compliance with native tissue is increasingly recognised as a determinant of regenerative outcome, as scaffolds with elastic modulus matched to the rabbit urethra (approximately 25 kPa) avoid both the over-rigid scarring response of stiffer constructs and the under-supporting failure of more compliant ones (Jin et al., 2025); quantification and tuning of the CPC scaffold’s elastic modulus to native urethral specifications would represent a parallel refinement axis. Modulation of the chitosan-to-PLGA mass ratio offers a further design lever for tuning the balance between mechanical integrity and bioactive surface chemistry. Chitosan fiber stability offers a further tuning axis: physical crosslinking (e.g., thermal treatment) or chemical crosslinking with agents such as genipin can slow chitosan resorption, potentially extending the bioactive window of the surface layers (Dash et al., 2011). Biofabrication approaches, including 3D and 4D printing of structured architectures, microfluidic gradient delivery of growth factors, and decellularized-ECM functionalization, represent emerging strategies for orchestrating spatially organized vascularization within tissue-engineered urethral constructs (Ławkowska et al., 2023). Translational evaluation will ultimately require larger animal models with urethral anatomy and healing physiology closer to humans, longer follow-up sufficient to capture late stricture and contracture, and head-to-head comparison with the autologous buccal mucosa graft that remains the clinical reference standard.

## 5 Conclusion

The first *in vivo* evaluation of a trilayer chitosan/PLGA/chitosan composite electrospun scaffold in a rabbit dorsal onlay urethroplasty model demonstrates that the construct is feasible to fabricate and implant, is well tolerated, and supports complete re-epithelialization, a controlled foreign-body response, and preserved urethral patency at 12 weeks. The trilayer composite did not significantly increase vessel density compared with monolayer PLGA at 12 weeks, and no superior regenerative or angiogenic performance was demonstrated. A non-significant trend toward lower macrophage scores in CPC was observed, which is hypothesis-generating and requires direct polarization phenotyping for confirmation. These findings support continued preclinical development of composite electrospun scaffolds for urethral reconstruction, with earlier endpoints, architecturally matched controls, and degradation characterization identified as priorities for future work.

## Data Availability

The raw data supporting the conclusions of this article will be made available by the corresponding author on reasonable request.
