# Supplementary material for: In vivo evaluation of a chitosan/PLGA/chitosan composite versus monolayer PLGA electrospun scaffold for dorsal onlay urethroplasty: a controlled study in rabbits
Source: Front Bioeng Biotechnol. 2026 Oct 19;14:1891449. doi: 10.3389/fbioe.2026.1891449 (PMC13724350; doi:10.3389/fbioe.2026.1891449)
Supplement: Supplementary file 1 [file Supplementaryfile1.docx]

### SUPPLEMENTARY

| SCORE | CPC (n=8) | PLGA (n=8) | SHAM (n=7) |
| --- | --- | --- | --- |
| MACROPHAGE SCORE |  |  |  |
| 0 | 1 | 0 | 0 |
| 1 | 4 | 0 | 1 |
| 2 | 0 | 1 | 4 |
| 3 | 2 | 6 | 1 |
| 4 | 1 | 1 | 1 |
| GIANT CELL SCORE |  |  |  |
| 0 | 2 | 2 | 6 |
| 1 | 2 | 2 | 1 |
| 2 | 1 | 0 | 0 |
| 3 | 3 | 2 | 0 |
| 4 | 0 | 2 | 0 |
| PMN SCORE |  |  |  |
| 0 | 6 | 5 | 4 |
| 1 | 1 | 3 | 2 |
| 2 | 1 | 0 | 0 |
| 3 | 0 | 0 | 1 |
| LYMPHOCYTE SCORE |  |  |  |
| 0 | 1 | 0 | 1 |
| 1 | 4 | 4 | 2 |
| 2 | 2 | 4 | 1 |
| 3 | 1 | 0 | 3 |
| PLASMA CELL SCORE |  |  |  |
| 0 | 8 | 6 | 7 |
| 1 | 0 | 2 | 0 |

**Supplementary Table S1. Distribution of inflammatory cell scores by treatment group.** Distribution of semi-quantitative inflammatory cell scores (0–4) across treatment groups in animals completing the planned 12-week follow-up. Values are presented as absolute counts for each score category.

**Supplementary Figure S1. Foreign-body findings in scaffold-treated animals at 12 weeks. (A)** H&E at higher magnification: white arrows indicate a focal cellular reaction with possible scaffold remnant (left) and a blue triangular structure consistent with polypropylene suture remnant (right) in the same field.
**(B)** H&E at lower magnification from a different animal: white arrow indicates a suture remnant within the region of interest (yellow outline).
On pathology review, the majority of amorphous foci across the cohort were interpreted as suture-related; confirmed residual scaffold polymer was rare and not unequivocally distinguishable from suture material on H&E alone (Section 3.6). Scale bars: 50 μm (A), 250 μm (B).

 **Supplementary Figure S2. Gross morphology of excised urethral specimens at the 12-week endpoint.** For each treatment group, a representative whole specimen is shown from two opposing views: (A, B) CPC, (D, E) PLGA, and (G, H) sham. Serial transverse cross-sections through the implantation site and adjacent native urethra are shown in (C) CPC, (F) PLGA, and (I) sham. The red box in (C) highlights a cross-section illustrating focal mucosal fibrosis and thickening at the region of interest. No macroscopic fistulae, necrosis, or calculi were observed in any specimen.
